# Validation of a simple screening tool for early diagnosis of advanced Parkinson’s disease in daily practice: the CDEPA questionnaire

**DOI:** 10.1038/s41531-018-0056-2

**Published:** 2018-07-02

**Authors:** Pablo Martinez-Martin, Jaime Kulisevsky, Pablo Mir, Eduardo Tolosa, Pilar García-Delgado, María-Rosario Luquin

**Affiliations:** 1 Centro de Investigación Biomédica en Red sobre Enfermedades Neurodegenerativas (CIBERNED), Madrid, Spain; 2 National Center of Epidemiology, Carlos III Institute of Health, Madrid, Spain; 3 Department of Neurology, Movement Disorders Unit, Hospital de la Santa Creu i Sant Pau (IIB Sant Pau), Universitat Autònoma de Barcelona, Universitat Oberta de Catalunya (UOC), Barcelona, Spain; 4 Unidad de Trastornos del Movimiento, Servicio de Neurología y Neurofisiología Clínica, Instituto de Biomedicina de Sevilla, Hospital Universitario Virgen del Rocío/CSIC/Universidad de Sevilla, Seville, Spain; 5 Movement Disorders Unit, Department of Neurology, Hospital Clínic de Barcelona, Institut d’Investigacions Biomèdiques August Pi i Sunyer (IDIBAPS), University of Barcelona, Barcelona, Spain; 6 Grupo de Investigación en Atención Farmacéutica, Facultad de Farmacia, Universidad de Granada, Granada, Spain; 7 Department of Neurology, Clínica Universidad de Navarra, Universidad de Navarra, Pamplona, Spain

## Abstract

Early clinical diagnosis of advanced Parkinson’s disease (APD) may be difficult. This study aimed to validate a simple screening tool, the CDEPA questionnaire (“Cuestionario De Enfermedad de Parkinson Avanzada” [Questionnaire for Advanced Parkinson’s Disease]), for the identification of APD in daily practice. The study included 173 consecutively selected patients with PD (40% were women, mean age was 68.4 ± 10.5 years), stratified according to the Hoehn and Yahr (HY) scale. The CDEPA questionnaire defined APD as the presence of severe disability requiring help for activities of daily living (ADL), motor fluctuations with limitation or inability to perform ADL, severe dysphagia, recurrent falls, or dementia. The diagnostic performance of the questionnaire was assessed against the gold standard criterion based on clinical judgment. PD was categorized as advanced in 65 (38%) patients when using the gold standard and in 109 (63%) patients when the CDEPA questionnaire was used. The CDEPA questionnaire and the gold standard agreed moderately (kappa statistic of 0.48, *P* < 0.001). The CDEPA classified APD with a sensitivity of 97%; specificity of 57%; total accuracy of 72.3%; and area under the curve (for a binary classifier) of 77.2%. Significant differences were found between the groups created by the CDEPA in several usual PD evaluations (HY Scale, SCOPA Motor Scale, Non-motor Symptoms Scale for PD, Clinical Impression of Severity Index for PD, Clinical Global Impression–Severity Scale, and Patient Global Impression–Severity Scale). CDEPA showed satisfactory inter-rater agreement (kappa = 0.88) and test–retest concordance (kappa 0.83). In conclusion, the CDEPA questionnaire is a valid, reliable, and useful instrument for easily screening APD.

## Questionnaire aids diagnosis of advanced disease

A Spanish questionnaire for advanced Parkinson’s disease, CDEPA, is a reliable tool for identifying patients with late-stage PD. Patients with advanced PD have severe motor and non-motor symptoms, and show poor response to conventional therapies. Early diagnosis is the key to determine which patients will benefit from the alternative treatment options such as deep brain stimulation. A study led by Pablo Martinez-Martin at the Carlos III Institute of Health in Madrid compared the accuracy of advanced PD diagnosis using the CDEPA questionnaire and a neurologist’s clinical judgment. The diagnostic accuracy of the questionnaire was >70% and the questionnaire results agreed with the diagnosis of advanced PD based on clinical judgment in 97% of cases. This quick and easy-to-administer questionnaire could be highly valuable for detecting advanced PD in outpatient clinics.

## Introduction

Affecting ~1% of individuals older than 60 years, Parkinson’s disease (PD) often spans decades of a patient’s lifetime.^1^ While PD has traditionally been considered as a motor disorder, it is now recognized as a complex condition with diverse clinical features that include neuropsychiatric and other non-motor manifestations in addition to its motor symptomatology.^2^ The term advanced PD (APD) is applied indistinctively to patients with long disease duration (independently of clinical manifestations) or with motor fluctuations and moderate or severe dyskinesia with disorders of gait and equilibrium, or with neuropsychiatric symptoms or cognitive impairment.^3,4^ In all these cases, motor and non-motor symptoms are associated with loss of independence and autonomy. At this stage, many symptoms do not improve with conventional therapies, and other alternatives such as deep brain stimulation (DBS), continuous subcutaneous apomorphine infusion, or continuous infusion of levodopa/carbidopa intestinal gel are required.^5,6^ Therefore, it is relevant to determine the patients’ clinical characteristics that can define APD and that make these patients eligible or ineligible for advanced therapies.

In a previous prospective nationwide and after a three-round Delphi study in Spain, the CDEPA questionnaire (Cuestionario De Enfermedad de Parkinson Avanzada [Questionnaire for Advanced Parkinson’s Disease]) were developed.^7^ This neurologist-based questionnaire is a simple screening tool that could be useful to identify patients with APD in the clinical setting. The presence of any definitive symptom (severe disability means requiring help for activities of daily living (ADL), presence of motor fluctuations with limitations to perform basic ADL without help, severe dysphagia, recurrent falls, or dementia) classifies the patient as APD. Probable and possible levels of certainty for motor and non-motor symptoms are also established (Table 1).

**Table 1:** Diagnostic criteria of advanced Parkinson’s disease using the CDEPA questionnaire

Domain	Level of certainty of the symptoms		
	Definitive^a^	Probable^b^	Possible^c^
General characteristics		Evolution time (around 10 years)	
Disability	Requiring help to perform daily living activities	Limitation to perform basic activities, although not requiring help	
Treatment-related motor symptoms	Motor fluctuations with an off time >25%, with limitation to perform the basic activities without requiring help	Functional disability due to dyskinesias with an on time >25%	
Disease-related motor symptoms	Severe dysphagia Recurrent falls	Moderate dysphagia Freezing of gait Moderate–severe dysarthria	Postural and equilibrium disorders
Disease-related non-motor symptoms			Symptomatic dysautonomia, including orthostatic symptomatic hypotension, excessive daytime somnolence
Neuropsychiatric and cognitive symptoms	Dementia	Hallucinations without preserved insight	Moderate–severe apathy Chronic presence of hallucinations with preserved insight Psychotic symptoms Mild cognitive impairment

All symptoms are scored as either “yes” (presence) or “no” (absence) [7]

^a^The presence of a definitive symptom makes the diagnostic of advanced Parkinson’s disease

^b^The association of two symptoms from different areas (general characteristics, disability, motor symptoms related to treatment, etc.) of the probable level places the case as definitive

^c^The association of one motor or non-motor symptom from the disease-related areas plus one symptom of the neuropsychiatric and cognitive areas in the possible level places the case as probable

The aim of this study was to validate the CDEPA questionnaire. Following completion of validation of the CDEPA in Spanish, a cross-cultural adaptation and validation in other languages would be useful to make this instrument available to other researchers and to compare data from different samples and backgrounds.

## Results

A total of 173 patients with PD (40.4% women) with a mean age of 68.4 ± 10.5 years were included in the study. The distribution by HY stage was stage 1, 9.4%; stage 2, 33.3%; stage 3, 29.8%; stage 4, 22.2%; and stage 5, 5.3%. Most patients were retired (61%) and had primary (47.9%) or secondary (23.7%) education. The mean age at PD onset was 58.2 ± 10.4 years, at PD diagnosis was 59.1 ± 10.3 years, and at the beginning of treatment for PD was 59.7 ± 10.2 years. The mean duration of PD was 10.3 ± 5.8 years. Descriptive data for the assessments applied in the study are shown in the Supplementary material.

The number of patients diagnosed with APD and non-advanced PD by the CDEPA instrument compared to clinical judgment (gold standard) is shown in Table 2. Clinical judgment correlated significantly with HY stage (*ρ* = 0.78, *P* < 0.001). According to the CDEPA questionnaire, advanced PD was present in 109 (63.0%) patients, whereas clinical judgment categorized 65 (37.6%) patients as having APD. The degree of agreement in the diagnostic accuracy of APD between the CDEPA questionnaire and the gold standard based on clinical judgment was moderate (kappa statistic 0.48, *P* < 0.001). The CDEPA instrument classified APD versus non-advanced PD with a sensitivity of 96.9% (63/65 cases) and a specificity of 57.4%. The positive and negative predictive values were 57.8% and 96.9%, respectively. The rate of false positives and false negatives were 42.6% and 3.1%, respectively. The diagnostic accuracy of the CDEPA questionnaire was 72.3%. The area under the curve (ROC analysis for binary classifiers) was 77.2%.

**Table 2:** Classification of patients in the advanced and non-advanced groups according to the CDEPA instrument versus clinical judgment

CDEPA instrument	Clinical judgment (gold standard)		Total
	Advanced PD	Non-advanced PD	
Advanced PD	63	46	109 (63.0)
Non-advanced PD	2	62	64 (37.0)
Total	65 (37.6)	108 (62.4)	173 (100)

No. (%)

Sensitivity was 96.9%; specificity was 57.4%; false positives were 42.6%; false negatives were 3.1%; positive predictive value was 57.8%; negative predictive value was 96.9%; total accuracy was 72.3%

As shown in Table 3, in all cases where the CDEPA questionnaire classified patients as APD, scores of the study rating scales (HY, S-MS, NMSS, and CISI-PD) were significantly higher than in patients classified as non-advanced.

**Table 3:** Diagnosis established by the CDEPA instrument and scores of the study assessments

	Diagnosis with the CDEPA questionnaire		
	Advanced mean ± SD (95% CI)	Non-advanced mean ± SD (95% CI)	*P* value
Hoehn and Yahr stage	3.3 ± 0.9 (3.1–3.5)	2.0 ± 0.7 (1.8–2.2)	0.001
SCOPA motor scale			
Examination (motor evaluation)	16.1 ± 6.4 (14.8–17.3)	6.6 ± 4.1 (5.6–7.7)	0.001
Activities of daily living	10.3 ± 4.5 (9.4–11.2)	2.9 ± 2.6 (2.2–3.5)	0.001
Dyskinesias	2.2 ± 1.9 (1.8–2.6)	0.5 ± 1.2 (0.1–0.8)	0.001
Motor fluctuations	2.7 ± 1.6 (2.4–3.0)	0.6 ± 1.1 (0.4–0.9)	0.001
Total scores	30.8 ± 11.72 (28.6–33.0)	10.6 ± 7.1 (8.8–12.35)	0.001
Non-motor symptoms scale			
Cardiovascular	1.2 ± 2.2 (0.8–1.7)	0.5 ± 1.3 (0.2–0.9)	0.003
Sleep/fatigue	11.4 ± 8.7 (9.8–13.1)	5.3 ± 5.7 (3.9–6.7)	0.001
Mood/cognition	12.4 ± 15.3 (9.5–15.3)	4.8 ± 11.2 (2.0–7.6)	0.001
Perceptual problems/hallucinations	1.9 ± 4.3 (1.1–2.7)	0.4 ± 1.8 (−0.1−0.8)	0.001
Attention/memory	5.9 ± 8.0 (4.4–7.4)	2.6 ± 5.1 (1.3–3.9)	0.001
Gastrointestinal tract	5.8 ± 6.3 (4.6–7.0)	2.3 ± 3.3 (1.5–3.1)	0.001
Urinary function	9.8 ± 8.7 (8.1–11.4)	4.8 ± 6.6 (3.2–6.4)	0.001
Sexual function	4.4 ± 6.1 (3.2–5.5)	3.2 ± 5.8 (1.8–4.7)	0.457
Miscellaneous	8.2 ± 7.5 (6.8–9.6)	4.8 ± 4.9 (3.6–6.0)	0.005
Total score	60.9 ± 41.0 (53.1–68.7)	29.0 ± 29.6 (21.4–36.2)	0.001
Clinical Impression of severity index for PD	13.0 ± 4.0 (12.2–13.8)	5.1 ± 3.2 (4.3–6.0)	0.001

When the characteristics of patients classified as true positives (TP) (diagnosed as APD by the CDEPA questionnaire and the gold standard) (*n* = 63) and false positives (FP) (classified as APD by the CDEPA questionnaire but as non-advanced PD by the gold standard) (*n* = 46) were compared, patients in the TP group were significantly older, had a more advanced HY stage and duration of disease, and showed higher scores in motor evaluation, ADL, and motor fluctuations of the S-MS scale, as well as higher scores in the domains of perceptual problems/hallucinations, attention/memory, gastrointestinal tract, and total score of the NMSS scale. All these differences were statistically significant. Also, significant differences between the groups of TP and FP in the CISI-PD, CGIS, and PGIS were observed, suggesting a more severe disease in the TP group.

Regarding CDEPA reliability, the inter-rater agreement for 96 patients was 95.8% and kappa was 0.88 (95% CI: 0.75–1.0). Furthermore, the test–retest concordance for 46 patients was 93.5% with a kappa equal to 0.83 (95% CI: 0.64–1.0).

## Discussion

Identification of disease severity in PD patients on clinical grounds in daily practice is important to outline a rational treatment plan in order to obtain maximum benefit from conventional therapies or to recommend alternative therapies in patients with late-stage PD. However, agreement among neurologists regarding the clinical characteristics that patients with APD should exhibit has not yet been reached.^3^ Late-stage PD is dominated by loss of autonomy due to motor and non-motor symptoms with a decrease in levodopa response,^8^ but studies on APD are limited.

In a previous study carried out by our group, a consensus on the definition of APD using a three-round Delphi methodology was reached.^7^ APD was defined as the disease stage in which certain symptoms and complications are present, with a detrimental influence on the overall patient’s health condition and with a poor response to conventional treatments. Definitive symptoms were considered for those whose presence, even when isolated, were enough to classify PD as APD, which included severe disability, motor fluctuations with limitation or inability to perform ADL without assistance, severe dysphagia, recurrent falls, or dementia. Probable and possible levels of certainty were also established (Table 1). In the studies performed by Coelho et al.,^9,10^ including the Barcelona and Lisbon cohort of 50 patients, postural instability causing frequent falls and prominent dysphagia were found to dominate the motor syndrome in late-stage PD. Dementia was also present in 50% of the patients in whom lack of tremor and absence of depression were independently associated with dementia. In addition, a high handicap score in this cohort measured by the London Handicap Score was mostly driven by the presence of dementia.^10^

Because a criterion like that defined in the CDEPA questionnaire has not been found in previously published studies, it was proposed to accept the neurologist’s clinical judgment as the best available benchmark (gold standard) to determine the presence or absence of APD. Clinical judgment (Table 4) was established after the clinician had thoroughly examined the patient using a battery of validated scales for severity evaluation in PD.^11,12^ Adequacy criteria for the selection of clinical judgment as the gold standard was supported by a high-positive correlation with HY stage classification.

**Table 4:** Parkinson’s disease (PD) clinical stage based on the neurologist’s clinical judgment (gold standard)

Clinical stage	Clinical characteristics
Initial^a^	Very mild clinical manifestations; absence of disability; and no need of pharmacologic treatment or only initial therapy at low effective doses
Mild^a^	Mild clinical and functional manifestations; minimal or no complications; and satisfactory response to conventional pharmacologic therapy
Moderate^a^	Moderate clinical manifestations and disability; good response; some complications are present, although not satisfactory to the conventional pharmacologic therapy
Advanced^b^	Severe clinical manifestations and disability; severe motor and non-motor complications; partial poor response to conventional pharmacologic therapy
Late-stage^b^	Very severe clinical manifestations and disability; very severe motor and non-motor complications; no response to conventional pharmacologic therapy

^a^Non-advanced PD

^b^Advanced PD

The ability of the questionnaire to distinguish between advanced and non-advanced PD was assessed by comparison with the gold standard. The sensitivity of the CDEPA questionnaire for the identification of APD was very high. In 97% of cases in which the gold standard indicated that the patient had APD, the CDEPA questionnaire coincided. The specificity was 57.4%. Misclassfication occurred in 48 cases (22.7%) (46 false positives and 2 false negatives). However, statistically significant differences were found between scores of all measures in the study using the groups of advanced and non-advanced PD identified by the CDEPA questionnaire. The performance of clinical judgment in relation to PD global severity evaluation according to the study assessments was also analyzed as a further measure of validity of the CDEPA instrument. Overall, the performance of the gold standard for the recognition of APD was similar to CDEPA. With regard to the CISI-PD, which has been shown to be the scale most closely correlated to any other variable measuring the severity of PD manifestations and functional disability,^12^ the cutoff point for the total CISI-PD score to discriminate advanced versus non-advanced patients according to the CDEPA questionnaire was 9. The equivalent cutoff point was 6 for the CGIS.

Discrepancy between the results of the CDEPA questionnaire and the gold standard was also explained when the characteristics of true positives and false positives were compared. It was observed that patients categorized as false positives were not as markedly affected as the patients categorized as true positives, suggesting that (1) they actually could be false positives for advanced PD because their disease is not advanced or (2) a greater subtleness than the “routine” clinical criterion for detection of APD is required (which might be provided by CDEPA). In this case, they would not be false positives, but true cases identified by CDEPA. A similar situation was reported in the study conducted by Stacy et al.^13^ that identified wearing-off signs and symptoms, in which the self-assessment patient questionnaire was more sensitive (57.1%) than the clinical assessment question (29.4%). Most importantly, only 3% of the APD cases according to the gold standard were lost by the CDEPA, whose high sensitivity and negative predictive values characterize this questionnaire as a potentially powerful screening instrument for the detection of APD. In addition, the inter-rater and test–retest reliability were satisfactory.

Limitations of the study include a lack of comparable measures for the criteria assessed with the CDEPA instrument, a small sample size partly due to advanced therapies (DBS, apomorphine, and Duodopa) as an exclusion criterion, and findings restricted to Spanish patients.

In conclusion, the CDEPA questionnaire is quick and easy to administer, suggesting that it could be integrated in PD outpatient clinics as a useful tool for screening PD patients, particularly due to the high sensitivity observed when classifying the advanced stage of the disease. However, a large-scale study including more patients with PD will help provide more extensive data to support the external validity of this instrument.

## Methods

### Design

A cross-sectional, observational, and multicenter study was conducted between 1 January 2015 and 8 July 2016. This study was performed in the outpatient clinics of the Departments of Neurology from 24 hospitals throughout Spain. Neurologists from these centers attended to patients with PD and voluntarily accepted to take part in the study. The primary objective of the study was to analyze the discriminative capacity of the CDEPA questionnaire for the diagnosis of APD with the neurologist’s clinical judgment selected as the “gold standard.” For this reason, values ≥80% for sensitivity and negative predictive, and moderate or higher agreement with the gold standard were deemed acceptable.

The study protocol was approved by the Ethics Committee of the participating centers. The study was conducted in accordance with the standards of good clinical practice and the current revision of the Declaration of Helsinki. Written informed consent to participate was obtained from all patients. Personal data were anonymized.

### Study participants

We enrolled consecutive patients with PD with at least 2 years of evolution at any stage of the Hoehn and Yahr (HY) scale;^14^ patients of any age, gender, and mental condition; caregivers of those patients who met the aforementioned criteria, but had limited ability to follow the interview by themselves; and patients who signed the informed consent. Patients who received DBS or any surgery for advanced PD, continuous subcutaneous apomorphine infusion, or continuous infusion of levodopa/carbidopa intestinal gel were excluded as they had previously been classified as patients with advanced PD.

### Procedures and assessments

Data were recorded during a single routine clinical visit. Each patient was visited by two independent neurologists (neurologist #1 and neurologist #2). Neurologist #1 assessed the eligibility criteria and invited the patient to participate in the study. Those patients who signed the informed consent underwent a clinical examination and were administered a battery of general and specific tests including the HY staging scale,^14^ the Scale for Outcomes in PD (SCOPA) Motor Scale (S-MS),^15^ the Non-Motor Symptoms Scale (NMSS) for Parkinson’s disease,^16^ the Clinical Impression of Severity Index for Parkinson’s disease (CISI-PD),^17,18^ the Clinical Global Impression–Severity Scale (CGIS),^19^ and the Patient Global Impression–Severity Scale (PGIS).^20,21^

Briefly, the S-MS contains 21 items in three sections, with possible scores ranging from 0 to 42 for examination, 0 to 21 for ADL, and 0 to 12 for complications (0 to 6 for dyskinesias and 0 to 6 for fluctuations). The NMSS has 30 items and 9 domains: cardiovascular (2 items), sleep/fatigue (4 items), mood/cognition (6 items), perceptual problems/hallucinations (3 items), attention/memory (3 items), gastrointestinal tract (3 items), urinary function (3 items), sexual function (2 items), and miscellaneous (4 items). Each item is scored based on a multiple of severity (from 0 to 3) and frequency (from 1 to 4). The CISI-PD is based on the impression of the clinician about the severity of four outstanding PD aspects: motor signs, disability, motor complications, and cognitive status. Each item domain is scored from 0 (normal) to 6 (very severe), with a total score ranging between 0 and 24. The CGIS includes seven response options from 1 “normal, not at all ill” to 7 “among the most extremely ill patients” and the 6-point PGIS scores: 1 as “normal”, 2–3 as “mild”, 4 as “moderate”, and 5–6 as “severe”.

At the end of the interview, neurologist #1 classified PD as advanced or non-advanced (gold standard) and answered the question “In which stage do you consider that this patient can be categorized?” according to pre-established criteria (for initial, mild, moderate, advanced, and late-stage) defined by the research group (Table 4). Advanced PD was defined as “a phase with severe clinical manifestations, disability, and motor and non-motor complications with poor response to conventional pharmacologic therapy (Table 4).”

Thereafter, neurologist #2, who was blinded to the results of the interview and the clinical judgment of neurologist #1, administered the CDEPA questionnaire^7^ during an immediately subsequent clinical encounter and made a diagnosis of advanced or non-advanced PD based only on this questionnaire. Details on the contents of the CDEPA questionnaire are shown in Table 1.

Testing of the inter-rater CDEPA reliability was carried out by two neurologists who independently applied the questionnaire. A second application, to explore the test–retest reliability, was performed 2–4 weeks after the first application.

### Data collection

For each patient, the following data were recorded: age, gender; working status (employee, sick leave, retired); education level (no studies, primary studies, secondary studies, university); age at the onset of symptoms; age at diagnosis of PD; duration of PD; age at starting a specific treatment for PD; current treatment for PD; and other pharmacologic treatment. Also, HY stage and the results of the S-MS, NMSS, CISI-PD, CGIS, and PGIS questionnaires were recorded in the “on” state. Patients were finally classified as advanced or non-advanced PD according to the neurologist’s clinical judgment and results of the CDEPA questionnaire.

### Statistical analysis

The patients were selected by consecutive sampling over the study period. It was planned that 12 patients stratified by HY stages (stages 1 and 2, 2 patients; stage 3, 5 patients; stages 4 and 5, 5 patients) would be recruited by each participating neurologist. A difference of 15% between the expected 40% APD in moderate/severe HY and the identified 25% in the real world, 100 patients in HY 3–5 would reach a 0.91 power. Categorical variables are expressed as frequencies and percentages, and continuous variables as measures of central tendency. Categorical variables were compared with the Fisher’s exact test. Because of the ordinal origin and non-normal distribution of data, groups of continuous data were compared with the Mann–Whitney *U* test or the Kruskal–Wallis test. The relationship between two continuous variables was assessed with the Spearman’s rank-order correlation coefficient, rho (*ρ*). The validity of the CDEPA questionnaire for the diagnosis of APD was assessed by means of the characteristic 2 × 2 table in front of the gold standard (classification by neurologist #1). Sensitivity, specificity, and positive and negative predictive values were calculated. The agreement level between the CDEPA and the gold standard was checked by the kappa index, judging the results according to Landis and Koch scale^22^ (0.00–0.20: negligible, 0.21–0.40: weak, 0.41–0.60: moderate, 0.61–0.80: substantial, 0.81–1.00: almost perfect). Statistical significance was set at *p* < 0.05. CDEPA inter-rater and test–retest reliability were analyzed with the kappa statistic. Statistical analyses were performed by means of the Statistical Analysis Systems (SAS Institute, Cary, NC, USA) version 9.1.3 Service Pack.

### Data availability

The data sets generated during and/or analyzed during the current study are available from the corresponding author on reasonable request.

## Electronic supplementary material

Supplementary material
