# Supplementary material for: Validation of a simple screening tool for early diagnosis of advanced Parkinson’s disease in daily practice: the CDEPA questionnaire
Source: NPJ Parkinsons Dis. 2018 Jul 2;4:20. doi: 10.1038/s41531-018-0056-2 (PMC6028449; doi:10.1038/s41531-018-0056-2)
Supplement: Supplementary file 1 — Supplementary material [file 41531_2018_56_MOESM1_ESM.pdf]

Supplementary Material

Table 1S. Descriptive data of the study questionnaires

	No. patients (%)	Mean ± SD (95% confidence interval)
Hoehn and Yahr staging (n = 173)		
Stage 1	16 (9.4)	
Stage 2	57 (33.3)	
Stage 3	51 (29.8)	
Stage 4	38 (22.2)	
Stage 5	9 (5.3)	
SCOPA Motor Scale (n = 173)		23.32 ± 14.16 (21.19 – 25.44)
Examination (motor evaluation)		12.7 ± 7.2 (11.7-13.8)
Activities of daily living		7.6 ± 5.3 (7.0-8.4)
Dyskinesias		1.6 ± 1.9 (1.3-1.8)
Motor fluctuations		2.0 ± 1.8 (1.7-2.2)
Non-Motor Symptoms Scale (n = 173)		
Cardiovascular		1.0 ± 1.9 (0.7-1.3)
Sleep/fatigue		9.2 ± 8.3 (7.9-10.4)
Mood/apathy		9.5 ± 14.4 (7.4-11.7)
Perceptual problems/hallucinations		1.4 ± 3.6 (0.8-1.9)
Attention/memory		4.7 ± 7.2 (3.6-5.8)
Gastrointestinal tract		4.5 ± 5.6 (3.8-5.4)
Urinary function		7.9 ± 8.3 (6.7-9.2)
Sexual function		3.9 ± 6.0 (3.0-4.8)
Miscellaneous		6.9 ± 6.9 (6.0-8.0)
Total score		49.0 ± 40.2 (43.0-55.1)
Clinical Impression of Severity Index for PD (n = 169)		10.1 ± 5.3 (9.3-11.*9)
Clinical Global Impression of Severity (n = 196)		
Normal, not at all ill	0	
Borderline	6 (3.5)	
Mildly ill	38 (22.9)	
Moderately ill	66 (38.4)	
Markedly ill	36 (20.9)	
Severely ill	25 (14.5)	
Among the most extremely ill patients	1 (0.6)	
Patient Global Impression of Severity (n = 172)		
Normal	5 (2.9)	
Very mild	8 (4.7)	
Mildly	39 (22.7)	
Moderately	75 (43.6)	
Severe	38 (22.1)	
Very severe	7 (4.1)	
Clinical judgement of PD stage (gold standard) (n = 173)		
Non-advanced	108 (62.4)	
Advanced	65 (37.6)	
CDEPA questionnaire (n = 173)		
Non-advanced	64 (37.0)	
Advanced	109 (63.0)	
